# Proteome changes in a human retinal pigment epithelial cell line during oxidative stress and following antioxidant treatment

**DOI:** 10.3389/fimmu.2023.1138519

**Published:** 2023-04-19

**Authors:** R. Scott Duncan, Andrew Keightley, Adam A. Lopez, Conner W. Hall, Peter Koulen

**Affiliations:** ^1^ Vision Research Center, Department of Ophthalmology, University of Missouri – Kansas City, School of Medicine, Kansas City, MO, United States; ^2^ Department of Biomedical Sciences, University of Missouri – Kansas City, School of Medicine, Kansas City, MO, United States

**Keywords:** gene ontology, retinal pigment epithelium (RPE) cells, oxidative stress, tocopherol, tert-butyl hydroperoxide, proteomics

## Abstract

Age related macular degeneration (AMD) is the most common cause of blindness in the elderly. Oxidative stress contributes to retinal pigment epithelium (RPE) dysfunction and cell death thereby leading to AMD. Using improved RPE cell model systems, such as human telomerase transcriptase-overexpressing (hTERT) RPE cells (hTERT-RPE), pathophysiological changes in RPE during oxidative stress can be better understood. Using this model system, we identified changes in the expression of proteins involved in the cellular antioxidant responses after induction of oxidative stress. Some antioxidants such as vitamin E (tocopherols and tocotrienols) are powerful antioxidants that can reduce oxidative damage in cells. Alpha-tocopherol (α-Toc or αT) and gamma-tocopherol (γ-Toc or γT) are well-studied tocopherols, but signaling mechanisms underlying their respective cytoprotective properties may be distinct. Here, we determined what effect oxidative stress, induced by extracellularly applied tBHP in the presence and absence of αT and/or γT, has on the expression of antioxidant proteins and related signaling networks. Using proteomics approaches, we identified differential protein expression in cellular antioxidant response pathways during oxidative stress and after tocopherol treatment. We identified three groups of proteins based on biochemical function: glutathione metabolism/transfer, peroxidases and redox-sensitive proteins involved in cytoprotective signaling. We found that oxidative stress and tocopherol treatment resulted in unique changes in these three groups of antioxidant proteins indicate that αT and γT independently and by themselves can induce the expression of antioxidant proteins in RPE cells. These results provide novel rationales for potential therapeutic strategies to protect RPE cells from oxidative stress.

## 1 Introduction

Age related macular degeneration (AMD) is the most common cause of blindness in people over the age of 65 (1, 2). Oxidative stress is one of the major pathophysiological contributors to AMD and it causes retinal pigment epithelium (RPE) dysfunction and cell death prompting a need for antioxidant therapy and the clinical use of vitamin E for AMD treatment and prevention (3–6). Physiological adaptation of RPE cells to oxidative stress initiated by reactive oxygen species, prompted us to systematically determine what effect oxidative stress has on global protein expression in human telomerase transcriptase-overexpressing (hTERT) RPE cells (hTERT-RPE).

Antioxidants have been tested for their ability to prevent oxidative stress in multiple cell types, including RPE (7). Some components of vitamin E, such as tocopherols, are powerful antioxidants that can reduce oxidative damage in cells (8, 9). Alpha-tocopherol (α-Toc or αT) is the prototypical tocopherol found in vitamin E and is the most abundant diet-supplied tocopherol in serum and tissues (9). Gamma-tocopherol (γ-Toc or γT) is highly abundant in vitamin E containing foods but is not present at high levels in the blood and tissues due to first pass metabolism. γT is a more effective antioxidant than αT and therefore may be a superior antioxidant for therapeutic purposes (10). Tocopherols have been shown to activate signaling pathways that promote cytoprotection (6). This prompted us to identify proteins involved in the cellular antioxidant response elicited by exposure to conditions of oxidative stress, exposure to tocopherols and exposure to both.

We carried out a proteomic screen of proteins involved in the cellular antioxidant response in hTERT-RPE cells exposed to sublethal tBHP concentrations versus those exposed to vehicle (control). We also determined whether exposure to αT or γT, in the presence or absence of tBHP, altered the expression of antioxidant proteins. We identified 33 differentially expressed proteins involved in cellular antioxidant response between control and tBHP exposure conditions. We subdivided the proteins into three groups based on biochemical function: glutathione metabolism/transfer, peroxidases and redox-sensitive proteins involved in cytoprotective signaling. In addition, we determined the effect of both αT and γT alone, and in combination with tBHP, on expression of proteins involved in the cellular antioxidant response.

We found that tBHP, αT, γT and combinations of αT or γT with tBHP led to unique changes in antioxidant proteins in hTERT-RPE cells that provide drug targets for the development of novel therapeutic strategies against oxidative stress in RPE cells.

## 2 Materials and methods

### 2.1 Cell culture and treatments

Human telomerase reverse transcriptase-overexpressing RPE (hTERT-RPE) cells (ATCC, # CRL-4000) were cultured in DMEM:F12 (1:1) + 10% FBS + 10μg/ml gentamicin to full confluence for experiments. A 1 x 10^5^ cells/ml cell concentration was used as a starting plating density followed by growth for 5 – 7 days until full confluence was reached. Cellular morphology was observed before treatments and cells exhibited a polygonal (pentagonal and hexagonal) shape with a monolayer. Tocopherols, α-tocopherol and γ-tocopherol (Millipore-Sigma, Burlington, MA), were solubilized in DMSO at 100mM and diluted in media to 100μM final concentration. Cells were exposed to tocopherols for 24 hours prior to treatment with the 100μM tert-butyl hydroperoxide (tBHP), the oxidant, or water as a vehicle control for 24 hours. The six paired treatment groups (24h treatment 1 - 24h treatment 2) were vehicle-control, vehicle-tBHP, αT-control, γT-control, αT-tBHP and γT-tBHP, the same as in a previous study (10) (see **Supplemental Figure 1**). A tocopherol concentration of 100μM was selected based on previous reports (~25 – 32 μM vitamin E in serum) and calculations based on the daily recommendation dose of vitamin E at 400IU. Experiments were carried out in duplicate for biological replicates.

### 2.2 Sample preparation for Mass Spectrometry

The hTERT RPE cells from two experiments (biological replicates) were collected, pelleted, frozen and lysed in RIPA buffer containing DNase. Cell lysates were cleared by centrifuging at 10,000 x g for 5 minutes. Cell lysates were alkylated and processed with denaturing rinses and buffer exchange for trypsin digestion using Microcon^®^ Centrifugal filter units (#MRCF0R030) essentially as described previously (11). Approximately 100ug protein from each growth condition was reduced (10mM TCEP, ~100uL volume), then diluted with 8M urea, 100mM Tris-HCl pH~8.5 to ~700uL, then transferred in steps into the Microcon^®^ filter cartridges. After two 200uL washes (8M urea), the samples were alkylated (100mM chloracetamide), then washed two additional times (8M urea). After a buffer exchange to 100mM Ammonium Bicarbonate, 100uL of the same buffer containing 1.5ug Trypsin plus ArgC (Promega #V5073) and incubated overnight at 37C. The peptides were harvested by centrifugation, labeled with Tandem Mass Tags (TMT, Thermo Fisher Scientific), and multidimensional LC (mudpit) was conducted on both TMT mixtures (two experiments). Fractionation was conducted under Basic (pH 10) Reversed Phase conditions (BRP) on a 0.4mm (ID) column 10cm length, with BEH130-C18 (Waters Corporation) 3-micron particle size matrix. Fractions were collected during a 140-minute gradient, Buffer A: 10mM Ammonium Formate, pH 10.0, Buffer B: 95% Acetonitrile, 10% Buffer A, 10uL per minute flow rate. Gradient was started with 1% B initial conditions, to 20% B at 90 minutes, 34%B at 120 minutes. Resulting fractions were concatenated (12) to generate 12, or 18 fractions, which were dried in preparation for LCMS.

### 2.3 LCMS data acquisition and database searches/TMT quantitation

Mudpit fractions were analyzed on a Fusion Lumos Orbitrap MS with SPS MS3 quantitation of TMT reporter ions, and on a QExactive MS with MS2 quantitation of TMT reporter ions. LCMS data was collected for each fraction with a 90-minute standard acidic reversed phase gradient for SPS-MS3, or 110 minutes for MS2 data acquisition. Parameters for the SPS MS3 approach were MS1 FTMS @120k resolution, MS2 ITMS CID 35.0, MS3 (HCD 55.0) FTMS at @50k using multinotch. Parameters for MS2 data acquired on the QExactive included 35k resolution for MS scans, and 17.5k resolution for MS2 scans (HCD 30.0) with 120mz fixed first mass. Data files from all TMT mudpit fraction sets from both experiments were searched with Proteome Discoverer 2.5. Technical replicates: Experiment 1 was analyzed twice by 12 fraction mudpit, collecting SPS MS3 data for TMT quantitation. Experiment 2 was analyzed by mudpit 3 times, with one 12 fraction mudpit collecting SPS MS3 data, and two additional times by MS2 TMT quantitation (including one 12 fraction dataset, and one 18 fraction dataset). Database searches were conducted with Proteome Discoverer 2.5 with mostly default settings after selecting instrument/quantitation mode, allowing N-terminal modifications (Acetyl, Met-loss, or both), deamidation of Gln/Asn, with static modifications N-terminus and Lysine residues modified with TMT 6plex, and carbamidomethylation of Cysteine residues. Human proteome database Uniprot UP000005640, with 77895 protein sequences, (6/28/2021) and a contaminants database were included as target databases. The reversed sequence databases were searched (percolator node) for FDR calculations within Proteome Discoverer. The mass spectrometry proteomics data have been deposited to the ProteomeXchange Consortium *via* the PRIDE (13) partner repository with the dataset identifier PXD039513 and 10.6019/PXD039513.

### 2.4 Data collection, reconciling and organization

Raw data from Proteome Discoverer was exported as a csv file for use in other programs or csv file converted to a Microsoft Excel file for sorting and manual analyses. Contaminant proteins were removed from the data and missing ID data was added. Entries with multiple IDs or proteins with different names but the same IDs (Entrez) were also included and reconciled later in the analysis. The data were filed using the R mapping code, which maps the gene names, Entrez IDs and uniport IDs. The average protein abundance was calculated for each paired treatment group (vehicle-control, vehicle-tBHP, αT-control, γT-control, αγ) was used as the value for imputation of missing abundance values. The standard deviation was calculated for each entry and used to find proteins that were least changed between the treatment groups.

Using literature (PubMed) and database (GeneCards^®^, Entrez, Uniprot) searches, we determine whether proteins were known to be expressed in RPE cells and whether they could be suitable housekeeping proteins for normalization. The mean and geometric mean of selected housekeeping genes (β-actin (ACTB), glyceraldehyde phosphate dehydrogenase (GAPDH), Proteasome 26S Subunit Ubiquitin Receptor, Non-ATPase 4 (PSMD4), and Adaptor Related Protein Complex 2 Subunit Beta 1 (AP2B1)) was calculated and was compared to the average of all abundances to identify the optimal normalizing factor. The mean normalized abundance, variance and standard deviation was calculated for each entry in each treatment condition. We perform two tailed students t-test for samples with possible unequal variance. We calculate the p-value and t-test value. We do this for the treatment group ratio comparisons. A significance cutoff of 0.05, which is 0.025 per tail, was used. We calculated the percent difference of fold change and log2 fold change for use in the EnrichR and pathway analysis programs. This is done for each file before they are integrated by joining them in R.

The concise list was processed in R and EnrichR. The EnrichR program uses the fold change values and the official gene names to perform a Genome Wide Enrichment analysis on statistically significant values. Use the key pathway, “KEGG_2021_Human”, “GO_Molecular_Function_2021”, “GO_Biological_Process_2021”, “GO_Cellular_ Component_2021” databases.

**Figure 1:**
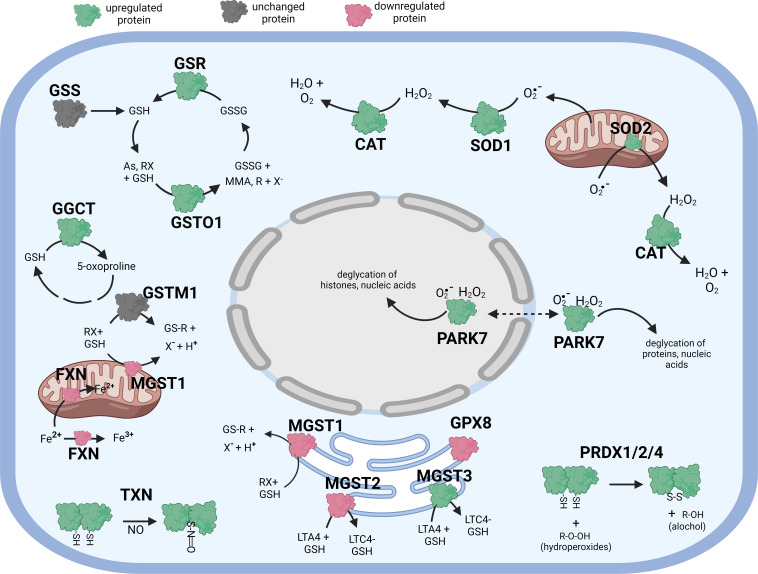
Diagramatic summary of the effect of tBHP exposure on antioxidant protein expression in hTERT-RPE cells. The antioxidant proteins focused on in this study are either upregulated (green), downreuglated (red) or unaffected (no change in expression) (grey) by oxidative stress. The arrows indicate the direction of a chemical reaction. Exposure of hTERT RPE cells to oxidant led to an increase in several proteins involved in glutathione homeostasis and transfer including GSR, GGCT, GSTO1 and MGST3. Conditions of oxidative stress also incerased the expression of PRDX1, PRDX2, PRADX4, SOD1, SOD2, CAT, TXN and PARK7. Figure created with BioRender.com.

**Figure 2:**
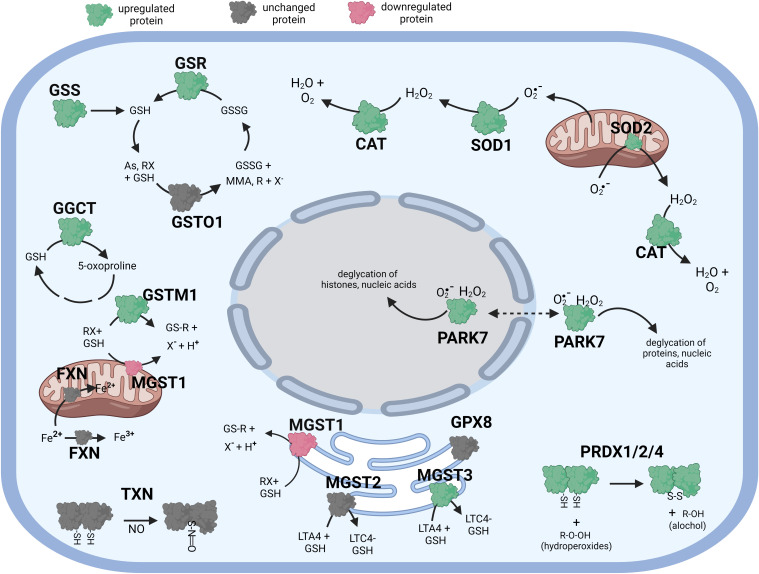
Diagramatic summary of the effect of αT exposure on antioxidant protein expression in hTERT-RPE cells. The antioxidant proteins focused on in this study are either upregulated (green), downregulated (red) or unaffected (no change in expression) (grey) by exposure to αT. The arrows indicate the direction of a chemical reaction. Exposure of hTERT RPE cells to αT led to an increase in several proteins involved in glutathione homeostasis and transfer including GSR, GGCT and MGST3. Exposure to αT also incerased the expression of PRDX1, PRDX2, PRADX4, SOD1, SOD2, CAT and PARK7. Figure created with BioRender.com.

**Figure 3:**
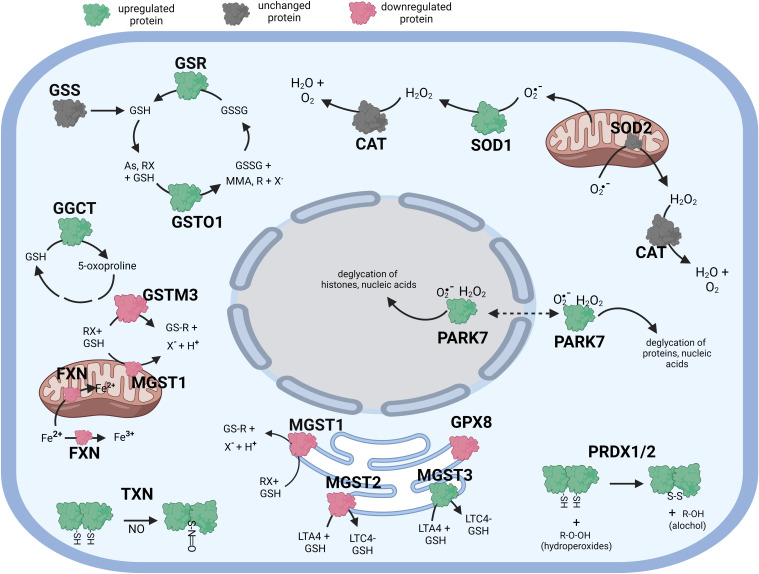
Diagramatic summary of the effect of γT exposure on antioxidant protein expression in hTERT-RPE cells. The antioxidant proteins focused on in this study are either upregulated (green), downreuglated (red) or unaffected (no change in expression) (grey) by exposure to γT. The arrows indicate athe direction of a chemical reaction. Exposure of hTERT RPE cells to γT led to an increase in several proteins involved in glutathione homeostasis and transfer including GSR, GGCT, GSTO1 and MGST3. Exposure to γT also incerased the expression of PRDX1, PRDX2, SOD1, TXN and PARK7. Figure created with BioRender.com.

**Figure 4:**
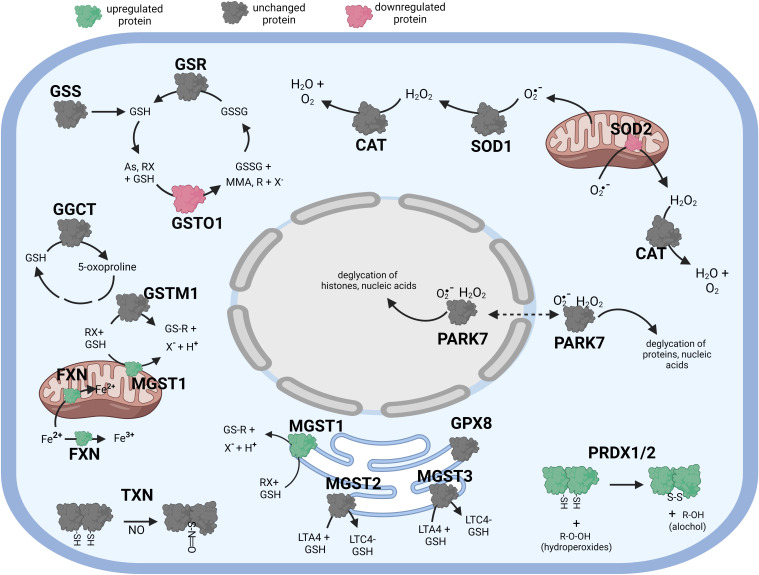
Diagramatic summary of the effect of αT pretreatment- tBHP post-treatmnet versus tBHP treatment alone on antioxidant protein expression. The antioxidant proteins focused on in this study are either upregulated (green), downreuglated (red) or unaffected (no change in expression) (grey) by exposure to αT. The arrows indicate athe direction of a chemical reaction. Exposure of hTERT RPE cells to αT followed by tBHP led to fewer upregulated antioxidant proteins compare to tBHP or αT alone, but there was an increase in MGST1, PRDX1, PRDX2 and FXN. Figure created with BioRender.com.

**Figure 5:**
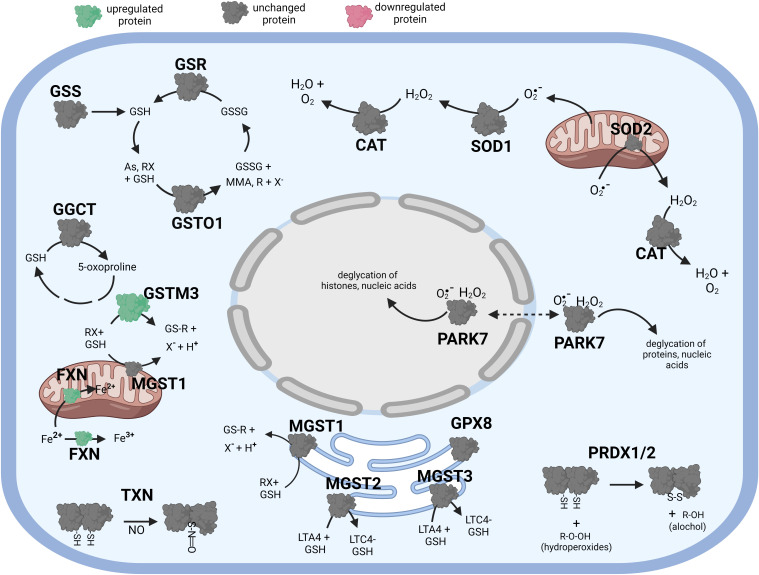
Diagramatic summary of the effect of γT pretreatment-tBHP post-treatement versus tBHP treatment alone on antioxidant protein expression. The antioxidant proteins focused on in this study are either upregulated (green), downreuglated (red) or unaffected (no change in expression) (grey) by exposure to αT. The arrows indicate athe direction of a chemical reaction. Exposure of hTERT RPE cells to γT followed by tBHP led to much fewer upregulated antioxidant proteins compare to tBHP or γT alone, but there was an increase in GSTM3 and FXN. Figure created with BioRender.com.

Pathway analysis and mapping to the KEGG pathway maps was carried out utilizing the R script we have written. The script takes the Entrez IDs and percent fold change difference, log_2_ fold changes, difference, or fold changes and filters the entries for significance and maps the entries to a designated KEGG pathway; this is often guided by the information from the enrichment test. The result is a colorized pathway map showing the genes identified in our data set and whether they are up or down regulated in our data. **Figures 1**–**5** were created with BioRender.com.

### 2.5 Gene ontology and functional category analyses

We used software-based analysis (R, EnrichR and KEGG) for gene ontology and pathway characterization and we manually group proteins based on broad function, such as glutathione function, peroxidase function and response to oxidative stress, that may not be identical to GO domain annotations. These manually groups were determined from curated gene/protein databases (GeneCards, Entrez, Uniprot) and literature.

We first sorted the data based on fold-change in expression and identified significantly up- and down-regulated proteins in the tBHP-exposed (oxidative stress) versus control (non-treated) group. From this list of 1,043 proteins, we manually screened and identified proteins known to be directly involved in antioxidant function in cells. To better determine whether a particular GO annotation was relevant to the proteins of interest, we selected the top 10 - 15 highest ranked GO categories (ranked by adjusted P value, odds ratio or by combined score) in each GO domain. We also prioritized GO categories in the ranked list with the narrowest (highly specific and descriptive) class (i.e., microsomal glutathione synthesis instead of glutathione metabolism). These narrow classes always have fewer proteins than broad classes, but they are associated with much better-defined function (or process or location).

We identified 33 significant differentially expressed proteins in response to oxidative stress and we subdivided the proteins into 3 groups based on the general protein functional class: proteins involved in glutathione metabolism/transfer, peroxidases/superoxide dismutases, and proteins that respond to oxidative stress (redox-sensitive proteins) or are involved in downstream functions such as cytoprotective signaling. We verified the relevance of each protein to antioxidant function by referencing gene/protein databases including GeneCards^®^, Entrez and Uniprot.

### 2.6 Statistical analysis

Relative protein abundance data for an experimental treatment group was normalized by dividing by the abundance of a control condition. Differences in the expression level of individual proteins between treatment groups were determined using a Student’s T-test in Microsoft Excel^®^. For the DCFDA assay on treated hTERT-RPE cells, differences were determine suing a one-way ANOVA using Graph Pad Prism^®^. Results were considered significant if the P-value was ≤0.05 (*) and highly significant is the P-value was ≤0.01 (**).

## 3 Results

### 3.1 Differentially Expressed proteins involved in the antioxidant response based on biological process gene ontology

We selected three broad molecular function categories for glutathione-related proteins:

1. glutathione binding/transfer
2. peroxidases and superoxide dismutases
3. proteins that respond to oxidative stress

We also uploaded the raw data into EnrichR for gene ontology (GO) analysis to identify biological process, molecular function and cellular component domains containing categories related to glutathione, peroxidase/superoxide dismutases and other proteins responsive to oxidative stress.

### 3.2 The effect of oxidative stress on the expression of proteins involved in glutathione metabolism and transfer

The treatment paradigm used for this study is summarized in **Supplemental Figure 1**. To induce oxidative stress in hTERT RPE cells, a sublethal concentration of tBHP was used to recapitulate a cellular antioxidant adaptive response where apoptosis and necrosis is negligible. The glutathione related GO biological process annotations enriched in the tBHP (oxidative stress) condition include, in order of highest to lowest score, ‘glutathione transport’, ‘glutathione derivative biosynthetic process’, ‘glutathione biosynthetic process’, ‘glutathione metabolic process’ and ‘glutathione transport’.

Of the 33 antioxidant proteins differentially expressed by tBHP-induced oxidative stress, six (6) were involved in glutathione metabolism or transfer. These include, in order from highest to lowest fold change in expression, gamma-glutamylcyclotransferase (GGCT), glutathione S-transferase omega 1 (GSTO1), microsomal glutathione S-transferase 3 (MGST3), microsomal glutathione S-transferase 2 (MGST2), glutathione peroxidase 8 (GPX8) and microsomal glutathione S-transferase 1 (MGST1) (**Table 1**). Five glutathione-related proteins were detected in the proteome but were not significantly altered, although two, glutathione S-transferase mu 3 (GSTM3) and glutathione peroxidase 1 (GPX1), showed a trend toward a decrease in expression (**Table 1**).

**Table 1:** Effect of tBHP on the expression of proteins involved in glutathione metabolism and transfer.

Protein	Fold-change	P value	Sig.
GSR	+17.7	0.0003	**
GGCT	+26.1	0.0003	**
GSTO1	+19.1	0.0013	*
GSS	+6.9	0.1672	n.s.
GSTM1	+3.0	0.8511	n.s.
GSTM2	+10.1	0.3216	n.s.
GSTM3	-14.1	0.0656	n.s.
MGST1	-35.3	0.0004	**
MGST2	-5.2	0.0008	**
MGST3	+15.1	0.0042	*
GPX1	-32.8	0.0653	n.s.
GPX8	-9.1	0.0073	*

* p≤ 0.05; **p ≤ 0.01; n.s., not significant.

Three proteins involved in glutathione metabolism were upregulated in response to oxidative stress: glutathione synthetase (GSS), glutathione reductase (GSR) and γ-glutamylcyclotransferase (GGCT). Exposure to tBHP led to the upregulation of GSR (+17.7-fold) and GGCT (26.1-fold), while GSS expression was not affected (**Table 1**). GSS is an ATPase that catalyzes the production of glutathione from γ-glutamylcysteine and glycine, while GSR is a flavoprotein responsible for reducing oxidized glutathione and therefore maintaining glutathione in a reduced state (14, 15). As such, the up-or down-regulation of GSS and GSR are critical for cellular detoxification and antioxidant activity. GGCT catalyzes the formation of pyroglutamic acid (also known as 5-oxoproline) from gamma-glutamyl dipeptides thereby playing a role in glutathione homeostasis (16). The upregulation of GSR and GGCT in response to oxidative stress suggests that oxidative stress may lead to improved glutathione homeostasis.

There were four (4) glutathione transferases that were differentially expressed in response to oxidative stress – 2 were up-regulated and 2 were down-regulated (**Table 1**; **Figure 1**). The remaining GST proteins were unaffected by oxidative stress. GSTO1 was upregulated by oxidative stress by 19.1-fold. GSTO1 is a homodimer found in the cytoplasm that exhibits thiol transferase activity and is also involved in the reduction of dehydroascorbate (vitamin C) (17, 18). The expression of other GST proteins such as the mu class GSTs (GSTM1 – GSTM3) were not significantly affected by oxidative stress, although there was a trend toward a decrease for GSTM3 (p = 0.066) (**Table 1**).

The last class of GST proteins that are differentially expressed in hTERT-RPE in response to oxidative stress are the membrane associated proteins in eicosanoid and glutathione metabolism (MAPEG) family (19). These include MGST1 – 6, but only MGST1 – 3 were detected in hTERT-RPE cells in this study. MGST3 was upregulated by oxidative stress by 15.1-fold while MGST1 and MGST2 were down regulated by 35.3- and 5.2-fold, respectively (**Table 1**; **Figure 1**). MGST3 is predominantly located in the endoplasmic reticulum and nuclear envelope. Like some mu class GST, some MGSTs like MGST2 and MGST3 are involved in prostaglandin and leukotriene formation (glutathione-conjugated forms).

The last differentially expressed protein related to glutathione function is glutathione peroxidase 8 (GPX8), which was down-regulated by 9.1-fold under conditions of oxidative stress. Glutathione peroxidase 1 (GPX1) was not affected by oxidative stress, although a trend toward a reduction in expression existed (p = 0.066) (**Table 1**). GPX1 has been detected in RPE cells (20), but to our knowledge, GPX8 expression in RPE has not been reported until now.

### 3.3 The effect of oxidative stress on peroxidases and superoxide dismutase function

SOD1 and SOD2 are two major antioxidant proteins present in most if not all cell types (21). Exposure of hTERT-RPE cells to tBHP led to an increase in SOD1 and SOD2 expression (+34.1- and 33.6-fold, respectively) (**Table 2**; **Figure 2**). SOD1 is a homodimeric cytoplasmic and nuclear protein involved in neutralizing free radicals, and mutations in SOD1 are associated with amyotrophic lateral sclerosis (ALS) type I (22). SOD2 is a homotetrameric protein expressed in mitochondria and is critical for neutralizing reactive oxygen species generated from the electron transport chain (23). Mutations in SOD2 are associated with premature aging and cardiomyopathies (24, 25). Catalase was upregulated 16.1-fold and PRDX4 was upregulated 8.4-fold by tBHP.

**Table 2:** Effect of tBHP on Peroxidase and SOD Expression.

Protein	Fold-change	P value	Sig.
SOD1	+34.1	2.8E-06	**
SOD2	+33.6	0.002	**
PRDX1	+26.9	0.009	**
PRDX2	+21.9	0.030	*
CAT	+16.1	0.005	**
PRDX4	+8.4	0.042	*
PRDX6	-0.05	0.269	n.s.
PRDX3	-3.9	0.578	n.s.
PRDX5	-15.2	0.139	n.s.

*p ≤ 0.05; **p ≤ 0.01; n.s., not significant.

Exposure of hTERT-RPE to tBHP led to a 26.9-fold and 21.9-fold upregulation in peroxiredoxin 1 (PRDX1) and peroxiredoxin 2 (PRDX2), respectively (**Table 2**; **Figure 1**). Peroxiredoxins thiol specific peroxidase enzymes that reduce peroxides including hydrogen peroxide and alkyl hydroperoxides and protect cells from oxidative stress (26–28). PRDX1 is expressed most highly in nucleus and extracellular space (little in endosomes/Golgi) while PRDX2 is expressed predominantly in the cytosol and may stabilize hemoglobin during oxidative stress 29, 30). PRDX3 and PRDX6 were unaffected by tBHP exposure (**Table 2**; **Figure 1**). PRDX4 is localized to the cytoplasm (extracellular > cytoplasm = nucleus) where it may play a role in NF-kappaB regulation (31, 32).

### 3.4 The effect of oxidative stress on other proteins involved in the cellular antioxidant response

Oxidative stress in hTERT-RPE cells led to changes in the expression of nine proteins involved in redox-sensitive signaling – two were involved in protein chaperone function (PARK7 and HYOU1), two were involved in heme/iron-sulfur transport (FXN and ABCB7), and the remaining five have various other roles.

PARK7 is upregulated 28.6-fold in hTERT-RPE exposed to tBHP. Park7 is a multi-functional redox-sensitive chaperone that has been shown to protect neurons from oxidative stress (33–37). Park7 can regulate NRF2, PINK1, the androgen receptor and NFκB pathways (34, 38). Mutations in PARK7 result in an early-onset form of Parkinson’s disease (39).

Park7 is a nucleotide and protein deglycase that can repair glycated proteins preventing the formation of advanced glycation end products (AGEs) (35, 37, 40). Can localize to the nucleus where it can deglycate histones (41). It is required for normal mitochondrial morphology and function and is involved in mitophagy (34, 39). Oxidative stress upregulated hypoxia upregulated protein 1 (HYOU1) 8.3-fold (**Table 3**; **Figure 1**). Hypoxia regulated protein 1 (HYOU1) is involved in ER chaperone and secretion functions. Although HYOU1 doesn’t appear to be involved in oxidative stress perse, it is a negative regulator of apoptosis and cellular protection during hypoxia (42, 43).

**Table 3:** Effect of tBHP on proteins involved in response to oxidative stress.

Protein	Fold-change	P value	Sig.
PARK7	+28.6	0.0007	**
TXN	+36.6	0.0002	**
HYOU1	+8.3	0.0029	**
FXN	-4.7	0.00003	**
HAGH	-5.6	0.0023	**
OXSR1	-5.6	0.0361	*
SIGMAR1	-11.8	0.1086	n.s.
TXNIP	-12.5	0.00001	**
ABCB7	-14.8	0.1166	n.s.
OXR1	-16.3	0.0214	*

*p ≤ 0.05; **p ≤ 0.01; n.s., not significant.

Exposure to oxidative stress led to a 36.6-fold increase in thioredoxin (TXN) and a 12.5-fold decrease in thioredoxin interacting protein (TXNIP). TXN is involved in nitric oxide-mediated protein nitrosylation at cysteine residues and can regulate caspase-3 activation (44–46). It can increase DNA binding of the redox-sensitive transcription factor AP-1 (47). TXNIP is a negative regulator of TXN.

Exposure of cells to tBHP led to a 4.7-fold reduction in frataxin (FXN) (**Table 3**). FXN is a protein located in mitochondria that is involved in the synthesis of heme and can transport Fe^2+^ into mitochondria and regulate the function of iron binding proteins involved in respiration (48). It can also generate Fe^3+^ *via* oxidation of Fe^2+^ (49). Trinucleotide expansions in the FXN gene can lead to Friedrich ataxia (50). Exposure of cells to tBHP led to a 14.8-fold decrease in ATP Binding Cassette Subfamily B Member 7 (ABCB7) expression (**Table 3**; **Figure 1**). ABCB7 is a transporter for heme (iron-sulfur clusters) from the mitochondria to the cytosol thereby maintaining metal homeostasis (51–53).

Oxidative stress downregulated oxidation resistance protein 1 (OXR1) 16.3-fold in hTERT-RPE cells. OXR1 is found in the nucleus and in mitochondria and is involved in neuronal protection against oxidative stress and subsequent apoptosis (54, 55). Exposure of hTERT-RPE to tBHP led to a 5.6-fold decrease in OXSR1 expression. Oxidative stress-responsive kinase 1 (OXSR1) is a serine-threonine kinase that is involved in regulating the phosphorylation of RELL proteins and MAPK14/P38α signaling (56, 57). To our knowledge, neither OXR1 nor OXSR1 presence/expression have been reported in RPE cells.

Exposure to tBHP led to an 11.8-fold decrease in sigma receptor 1 (SIGMAR1) expression (**Table 3**; **Figure 1**). SIGMAR1 protein is involved in ER lipid transport and regulation of plasma membrane lipid microdomains (58) ^56^. Involved in the regulation of different receptors it plays a role in BDNF signaling and EGF signaling. Also regulates ion channels like the potassium channel and could modulate neurotransmitter release. SIGMAR1 also appears to regulate some growth factor receptors and ion channels (59). It is also involved in several other cellular functions including cellular proliferation, cyto-protection against oxidative stress, mitochondrial transport, learning and memory (60).

### 3.5 The Effect of α-Tocopherol (αT) or γ-Tocopherol (γT) alone on antioxidant protein expression

Like cells exposed to tBHP, cells exposed to αT or γT exhibited an increase in GGCT (+12.3-fold for αT and +17.1-fold for γT), GSR (+14.5-fold for αT and +20.1-fold for γT) and MGST3 (+11.6 for αT and +11.5-fold for γT) (**Table 4**; **Figures 2**, **3**). Exposure to αT led to a 12.4-fold increase in GSS and a 19.3-fold decrease in GSTM1, while tBHP or γT had no effect (**Table 4**; **Figures 2**, **3**). Similar to tBHP exposure, αT or γT exposure increased the expression of SOD1 (+21.9- and +20.1-fold, respectively), PRDX1 (+23.9- and +35.0-fold, respectively), PRDX2 (+31.8- and +34.5-fold, respectively; **Table 4**) and PARK7 (+20.6- and +26.1-fold, respectively; **Table 5**).

**Table 4:** The effect of αT and γT on the expression of proteins involved in glutathione metabolism and transfer.

	α-T			γ-T		
Protein	Fold change	P value	Sig.	Fold change	P value	Sig.
GSR	+14.5	0.001	**	+20.1	0.001	**
GGCT	+12.3	0.021	*	+17.1	0.004	**
GSTO1	+8.9	0.095	n.s.	+11.0	0.038	*
GSS	+12.4	0.015	*	+3.5	0.516	n.s.
GSTM1	+13.9	0.006	**	-2.9	0.201	n.s.
GSTM2	+1.0	0.641	n.s.	+2.3	0.837	n.s.
GSTM3	-11.4	0.124	n.s.	-19.3	0.016	*
MGST1	-23.9	0.006	**	-21.1	0.012	*
MGST2	+2.5	0.514	n.s.	-10.7	0.007	**
MGST3	+11.6	0.006	**	+11.5	0.016	*
GPX8	+0.3	0.876	n.s.	-6.2	0.042	*

*p ≤ 0.05; **p ≤ 0.01; n.s., not significant.

**Table 5:** The effect of αT and γT on the expression of Per-oxidases, SOD and CAT.

	α-T			γ-T		
Protein	Fold Change	P Value	Sig.	Fold Change	P Value	Sig.
PRDX1	+23.9	0.017	*	+35.0	0.002	**
PRDX2	+31.8	0.005	**	+34.5	0.003	**
PRDX3	+18.8	0.460	n.s.	+16.7	0.551	n.s.
PRDX4	+18.5	0.010	**	+8.6	0.159	n.s.
PRDX5	+17.7	0.103	n.s.	+18.2	0.108	n.s.
PRDX6	-1.3	0.082	n.s.	-4.3	0.040	*
SOD1	+21.9	0.000	**	+29.1	0.000	**
SOD2	+18.9	0.044	*	+10.1	0.218	n.s.
CAT	+16.8	0.016	*	+9.6	0.123	n.s.

*p ≤ 0.05; **p ≤ 0.01; n.s., not significant.

PRDX4 was uniquely upregulated by αT alone but was unaffected by tBHP or γT (**Table 5**). GSTM3, PRDX6, SIGMAR1 and ABCB7 were uniquely downregulated by γT alone but were unaffected by tBHP or αT (**Tables 4**–**6**).

**Table 6:** The effect of αT and γT on the expression of proteins involved in the antioxidant response.

	α-T			γ-T		
Protein	Fold Change	P Value	Sig.	Fold Change	P Value	Sig.
PARK7	20.6	0.006	**	26.1	0.002	**
TXN	6.5	0.500	n.s.	44.2	0.000	**
TXNIP	-6.7	0.013	*	-5.0	0.033	*
FXN	-0.9	0.045	*	-4.6	0.000	**
SIGMAR1	-12.0	0.111	n.s.	-28.6	0.002	**
ABCB7	-9.6	0.365	n.s.	-20.9	0.021	*
OXR1	1.7	0.533	n.s.	-9.4	0.230	n.s.
OXSR1	-1.6	0.386	n.s.	-13.9	0.001	**
HYOU1	7.1	0.005	**	0.2	0.954	n.s.
HAGH	10.5	0.005	**	-1.1	0.577	n.s.

*p ≤ 0.05; **p ≤ 0.01; n.s., not significant.

Like cells exposed to tBHP, cells exposed to αT or γT exhibited a decrease in MGST1 (-23.9-fold for αT and -21.1-fold for γT). Exposure to αT did not reduce the expression in any of the AO proteins, but γT itself, reduced the expression of GSTM3 (-19.3-fold), PRDX6 (-4.3-fold), SIGMAR1 (-28.6-fold) and ABCB7 (-20.9-fold). These findings suggest that αT and γT share some, but not all mechanisms for AO protein upregulation.

Some proteins were similarly up- or down-regulated by tBHP and αT or tBHP and γT. For example, SOD2 and CAT were upregulated by tBHP (+33.6-fold for SOD2 and +16.1-fold for CAT) and γT (+18.9-fold for SOD2 and +16.8-fold for CAT) but were unaffected by αT. GSTO1 and TXN were upregulated by tBHP (+19.1-fold for GSTO1 and +36.6-fold for TXN) and γT (+11.0 for GSTO1 and +44.2-fold for TXN) but were unaffected by αT. Likewise, MGST2, GPX8, TXNIP, OXR1 and OXSR1 were downregulated by tBHP and γT but were unaffected by αT (**Tables 1**, **3**).

### 3.6 The effect of α-Tocopherol (αT) or γ-Tocopherol (γT) in combination with tBHP on antioxidant protein expression

We determined what effect αT or γT pre-exposure had on the ability of tBHP to induce AO protein expression. Several AO proteins that were upregulated by tBHP exposure were reduced by αT or γT pre-exposure, including SOD1, TXN, PRDX1 and PRDX2 (**Tables 7**–**9**; **Figures 4**, **5**). Some proteins that were upregulated by tBHP were significantly downregulated below baseline by αT, but not γT, pre-exposure, including SOD2 and CAT (**Table 8**). Like αT, pre-exposure to γT downregulated the tBHP mediated increase in CAT expression.

**Table 7:** The effect of αT or γT with tBHP on the expression of proteins involved in glutathione metabolism and transfer.

	GGCT	GSR	GSS	GSTO1	GSTM1	GSTM3	MGST1	MGST2	MGST3	GPX8
veh-tBHP/veh-NT	+26.1	+17.7		+19.1			-35.3	-5.2	+15.1	-9.1
αT-NT/veh-NT	+12.3	+14.5	+12.4		+13.9		-23.9		+11.6	
γT-NT/veh-NT	+17.1	+20.1		+11.0		-19.3	-21.1	-10.7	+11.5	-6.2
αT-tBHP/αT-NT			-5.9	-10.75	-24.4		+23.9	-6.0		
γT-tBHP/γT-NT						+16.4	-7.1			
αT-tBHP/veh-tBHP				-18.28			+45.8			
γT-tBHP/veh-tBHP						+9.5				

**Table 8:** The effect of tBHP, αT or γT, or αT or γT and tBHP on the expression of Peroxidases, SOD and CAT.

	PRDX1	PRDX2	PRDX3	PRDX4	PRDX5	PRDX6	SOD1	SOD2	CAT
veh-tBHP/veh-NT	+26.9	+21.9					+34.1	+33.6	+16.1
αT-NT/veh-NT	+23.9	+31.8		+18.5			+21.9	+18.9	+16.8
γT-NT/veh-NT	+35	+34.5				-4.3	+29.1		
αT-tBHP/αT-NT	+8.4		-35.3		-24.3	-15.2			
γT-tBHP/γT-NT		-8.5	-29.9		-23.3			+13.3	
αT-tBHP/veh-tBHP	+5.9	+11.0						-15.63	
γT-tBHP/veh-tBHP									

**Table 9:** The effect of αT or γT and tBHP on the expression of proteins involved in the antioxidant response.

	PARK7	TXN	TXNIP	FXN	SIGMAR1	ABCB7	OXR1	OXSR1
veh-tBHP/veh-NT	+28.6	+36.6	-12.5	-4.7			-16.3	-5.6
αT-NT/veh-NT	+20.6			-0.9				
γT-NT/veh-NT	+26.1	+44.2	-5.0	-4.6	-28.6	-20.9	-9.4	-13.9
αT-tBHP/αT-NT	-11.7	+22.7		-2.1	-15.1			-16.4
γT-tBHP/γT-NT		-10.0			+23.0	+17.4		+13.8
αT-tBHP/veh-tBHP				+1.9	-15.3		+21.2	
γT-tBHP/veh-tBHP				+1.5				

We also determined what effect tBHP exposure had on the ability of αT or γT to induce AO protein expression. Two AO proteins that were upregulated by αT or γT pre-exposure but were reduced by subsequent tBHP exposure were SOD1 and PRDX1 (**Table 8**). Similarly, exposure to tBHP was able to inhibit the induction of PRDX4 mediated by αT. Exposure to tBHP was able to prevent the upregulation of PRDX2 mediated by γT and it even caused a significant downregulation of PRDX2 below baseline. Exposure to tBHP was able to prevent the downregulation of PRDX3 and PRDX6 mediated by γT (**Table 8**).

We previously reported that αT, but not tBHP, exposure of hTERT-RPE cells induced the expression of Nrf2 but did not lead to the nuclear translocation of Nrf2 (10). Exposure to tBHP, on the other hand, had no effect on Nrf2 expression but it led to an increase in Nrf2 nuclear translocation, suggesting it had become activated.

We determined what effect αT or γT pre-exposure had on the ability of tBHP to induce AO protein expression. Some AO proteins that were upregulated by tBHP exposure were reduced by αT or γT pre-exposure include TXN and HYOU1. Some proteins that were upregulated by tBHP were significantly downregulated below baseline by αT, but not γT, pre-exposure, including PARK7.

We also determined what effect tBHP exposure had on the ability of αT or γT to induce AO protein expression. Several AO proteins that were upregulated by αT or γT pre-exposure were reduced by tBHP, including PARK7 and HYOU1 **Table 9**). Exposure to tBHP was able to inhibit the induction of TXN mediated by γT. Exposure to tBHP was even able to upregulate the expression of SIGMAR1 after the downregulation its by γT (**Table 9**). Since α-tocopherol and γ-tocopherol, themselves, could upregulate some AO proteins, we verified that this activity of tocopherols was not mediated by an unexpected increase in oxidative stress. We loaded cells with the redox-sensitive dye, DCFDA, and then treated cells with 100μM tBHP, 100μM α-tocopherol or 100μM γ-tocopherol for 2 hours. Exposure to tBHP led to an increase in DCFDA fluorescence by 4.7-fold, while neither αT nor γT had an effect on DCFDA fluorescence (**Supplemental Figure 2**). This suggests, as expected, that tBHP generates reactive oxygen species while tocopherol do not.

## 4 Discussion

In this study, we pretreated hTERT-RPE cells with αT or γT (or vehicle) followed by treatment with tBHP to induce oxidative stress (or treated with vehicle as a control). The treatment paradigm carried out in this study was a preventative or prophylactic approach rather than a post-injury intervention strategy. After tocopherol (or vehicle) treatment, we carried out a proteomics study on treated cells to determine the effect of tBHP and/or tocopherols on the expression of proteins involved in redox homeostasis and antioxidant function. Exposure of cells to tBHP to induce oxidative stress resulted in the upregulation and downregulation of several proteins involved in the cellular antioxidant response. Similarly, treatment of cells with αT or γT, in the presence or absence of tBHP, led to changes in antioxidant protein expression. In fact, some proteins upregulated under conditions of oxidative stress were also upregulated by exposure to tocopherols alone, suggesting that cells may be ‘primed’ to upregulate some antioxidant proteins in the absence of oxidant. This is the first study carried out where hTERT-RPE were exposed to sublethal oxidative stress to detect differential expression of antioxidant-related proteins. It is also the first study in hTERT-RPE cells detecting differential changes in antioxidant-related proteins in response to exposure to tocopherols.

At present is not clear how tocopherols exhibit similar effects as tBHP on the expression and/or activity of certain antioxidant response proteins as tocopherols and tBHP are chemically distinct. One possibility is that they may activate similar signaling pathways as tBHP, thereby leading to similar changes in AO protein expression. This may occur even though tocopherols and tBHP are structurally and chemically different. It is possible that tocopherols, despite having no pro-oxidant activity, act as agents that ‘pre-condition’ cells tor oxidative stress.

Previous studies describe proteomic analysis done on RPE, although several were carried out using the human ARPE-19 cell line, which does not recapitulate some of the properties of primary RPE cells (61, 62). In this study, we utilized hTERT RPE cells as a model system as they are easy to maintain in culture and they retain many of the phenotypic hallmarks of native RPE cells. Cellular morphology hTERT-RPE cells exhibited a polygonal (pentagonal and hexagonal) shape cells formed a monolayer. hTERT-RPE morphology was also observed after tBHP and tocopherol treatments to ensure no noticeable cellular changes occurred. None of the treatments caused a noticeable change in the hTERT-RPE cell morphology compared to vehicle-treated controls. Use of hTERT RPE cells also circumvent some of the challenges with growing primary RPE cells to densities sufficient for proteomic analysis. A previous proteomics study was carried out revealing that there was differential protein expression between hTERT-RPE and human primary RPE (63). While hTERT-RPE may not provide identical genotypic and phenotypic characteristics as native primary RPE, this cell line does provide many of the same key cell-specific protein markers and cellular processes as primary RPE.

Oxidative stress generated by tBHP upregulated several proteins involved in glutathione synthesis and transfer (i.e., GST family members). GGCT is involved in glutathione homeostasis and tBHP, αT and γT upregulated its expression. GST proteins are involved in the metabolism of xenobiotic compounds, drugs, and toxins, such as carcinogens. Some GSTM members are Involved in the generation of glutathione conjugates of prostaglandins A2 and J2 and prostaglandin J2 (PGA2 and PGJ2) (64). hTERT RPE cells expressed GSTO1, GSTM1 and MGST members. As expected, tBHP upregulated the expression of GSTO1 but, surprisingly, γT did as well.

Exposure of cells to tBHP reduced the expression of FXN, whereas neither αT nor γT had an effect. Since FXN is involved in heme assembly, maintenance of iron-sulfur cluster proteins and oxidation of Fe^2+^, its downregulation may be detrimental to combating oxidative stress. Likewise, tBHP exposure downregulates GPX8 expression which may make cells more susceptible to oxidative stress.

GSTM1 is expressed in RPE and RPE-derived cells such as ARPE-19 cells and GSTM1 gene copy number variations have been associated with AMD (65–67). Exposure of cells to αT, but not tBHP nor γT, led to the upregulation of GSTM1. MGST1 has been detected in RPE cells (68) and we detected it in our cell model system. Exposure of cells to tBHP, αT or γT led to the downregulation of MGST1.

Data on the molecular function of a protein can be determined experimentally or can be inferred based on amino acid sequence, secondary structure, presence of functional domains and similarity (in sequence and structure) to well-studied proteins. These parameters have high and accurate predictive value. The GO annotation ‘phospholipid-hydroperoxide glutathione peroxidase activity’ is very specific (narrow). ‘Peroxidase activity’ is broad as but isn’t specific for glutathione. Glutamate transport annotations, ‘ABC-type glutathione S-conjugate transporter activity’ and ‘transmembrane transporter activity – transports or maintains localization of S-(2,4-dinitrophenyl)glutathione’, are also present in the list and represent more specific functions.

The determination of a protein’s relevance to a biological process can be determined experimentally by use of inhibitors, genetic knock down or knock out of the protein. The relevance to a biological process can be inferred from the known molecular function of the protein and coevolutionary relationship between the emergence of the protein and emergence of the process.

The last domain, cellular component, is assigned the lowest priority because experimental methods that determine location/localization can be sensitive to methodological pitfalls. For example, it is often difficult to prevent fraction contamination during subcellular fractionation. Colocalization studies using microscopy are accurate only if the antibody used is highly specific for the proteins of interest (that is it has a very low level of cross reactivity). Lastly, co-immunoprecipitation experiments may provide a false positive result as proteins shown to interact within a cell lysate may not interact in an intact cell. Regardless of these methodological issues, the mere presence of a protein with a particular cell compartment or structure itself does not provide enough information about its function and relevance to a particular biological process.

Because γT itself reduced the expression of PRDX3, PRDX5, PRDX6 and GSTM3, it appears as if γT directly reduces the expression of these proteins. Although this might be the case, it is also conceivable that γT may be neutralizing ‘background’ or basal level of free radical formation that maintains the tonic expression of these proteins.

There are two possible explanations for how these proteins were upregulated or downregulated: an increase or decreases in basal gene expression or an increase or decrease in protein turnover mediated by proteasomal degradation. As such, we determined whether there were redox-sensitive transcription factor binding sites in the promoters for the upregulated gene products and if there were upregulated E3 ubiquitin ligases known to target the downregulated glutathione metabolic proteins. All detected E3 ubiquitin ligases were down regulated suggesting that proteasomal degradation may not be the main way the glutathione proteins were downregulated.

This study has identified several proteins involved in antioxidant function that are up- or down-regulated by oxidative stress and/or tocopherols. Furthermore, we determined the effect of tocopherol pretreatment on the effect of tBHP to alter antioxidant proteins expression as well as the effect of tBHP post-treatment on the effects tocopherols on antioxidant protein expression. This study provides useful information about what proteins may elicit a protective antioxidant response in RPE cells and which proteins may be therapeutic targets in AMD.

## Data availability statement

The mass spectrometry proteomics data have been deposited to the ProteomeXchange Consortium *via* the PRIDE partner repository (13) with the dataset identifier PXD039513 and 10.6019/PXD039513.

## Author contributions

PK conceived and designed the experiments. RD, AK, AL, CH and PK performed the experiments. RD, AK and PK wrote the manuscript. All authors contributed to the article and approved the submitted version.

## Supplementary material

Supplementary Figure 1 Tocopherol-tBHP Treatment Paradigm for hTERT RPE Cells. hTERT RPE cells were exposed for to either 100μM αT, 100μM γT or equivalent volume of DMSO control for 24 hours. After the 24-hour exposure, cells were rinsed and media containing either tBHP or vehicle control was added. Cells remained exposed to tBHP (or vehicle) for 24 hours.

Supplementary Figure 2 Comparison of reactive oxygen species (ROS) measurement between tBHP and tocopherols in hTERT-RPE cells. hTERT-RPE cells were loaded with DCFDA, a redox-sensitive fluorescent dye, for 1 hour, rinsed 3 times with PBS and exposed to either 100μM tBHP or 100μM αT or γT for 4 hours. Cells were read in a plate reader to detect fluorescence intensity. Exposure to tBHP led to a 5.8-fold increase in DCFDA fluorescence indicating an elevated presence of ROS while exposure to tocopherols had no effect on DCFDA fluorescence.
