# Supplementary figures and images for: Proteome changes in a human retinal pigment epithelial cell line during oxidative stress and following antioxidant treatment
Source: Front Immunol. 2023 Apr 19;14:1138519. doi: 10.3389/fimmu.2023.1138519 (PMC10154683; doi:10.3389/fimmu.2023.1138519)

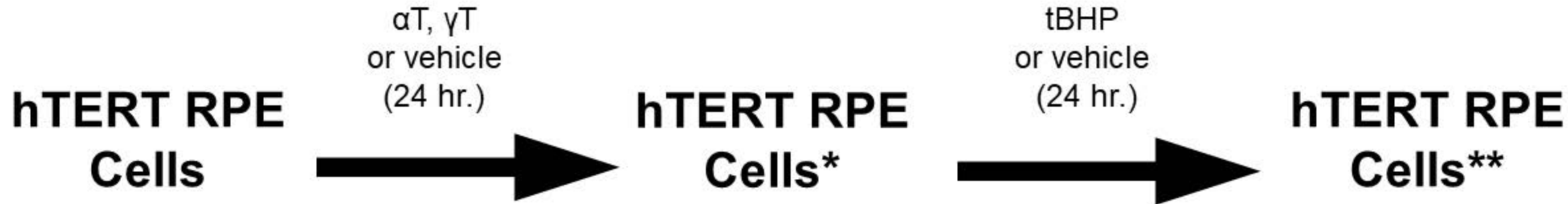

\* cells at 24 hrs.

\*\* cells at 48 hrs.

Supplement: Supplementary Figure 1 — Tocopherol-tBHP Treatment Paradigm for hTERT RPE Cells. hTERT RPE cells were exposed for to either 100μM αT, 100μM γT or equivalent volume of DMSO control for 24 hours. After the 24-hour exposure, cells were rinsed and media containing either tBHP or vehicle control was added. Cells remained exposed to tBHP (or vehicle) for 24 hours. [file Image_1.pdf]

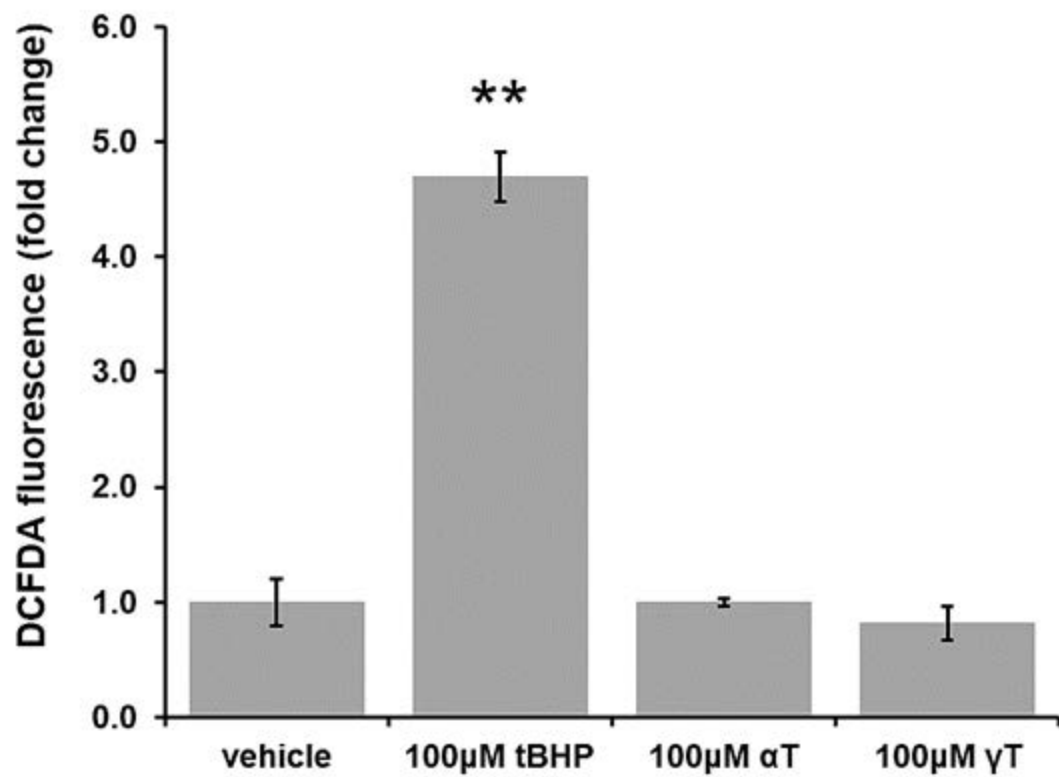

Supplement: Supplementary Figure 2 — Comparison of reactive oxygen species (ROS) measurement between tBHP and tocopherols in hTERT-RPE cells. hTERT-RPE cells were loaded with DCFDA, a redox-sensitive fluorescent dye, for 1 hour, rinsed 3 times with PBS and exposed to either 100μM tBHP or 100μM αT or γT for 4 hours. Cells were read in a plate reader to detect fluorescence intensity. Exposure to tBHP led to a 5.8-fold increase in DCFDA fluorescence indicating an elevated presence of ROS while exposure to tocopherols had no effect on DCFDA fluorescence. [file Image_2.pdf]
